# Bootstrap-based Support of HGT Inferred by Maximum Parsimony

**DOI:** 10.1186/1471-2148-10-131

**Published:** 2010-05-05

**Authors:** Hyun Jung Park, Guohua Jin, Luay Nakhleh

**Affiliations:** 1 Department of Computer Science, Rice University, 6100 Main Street, MS 132, Houston, Texas 77005, USA

## Abstract

**Background:**

Maximum parsimony is one of the most commonly used criteria for reconstructing phylogenetic trees. Recently, Nakhleh and co-workers extended this criterion to enable reconstruction of *phylogenetic networks*, and demonstrated its application to detecting reticulate evolutionary relationships. However, one of the major problems with this extension has been that it favors more complex evolutionary relationships over simpler ones, thus having the potential for overestimating the amount of reticulation in the data. An *ad hoc *solution to this problem that has been used entails inspecting the improvement in the parsimony length as more reticulation events are added to the model, and stopping when the improvement is below a certain threshold.

**Results:**

In this paper, we address this problem in a more systematic way, by proposing a nonparametric bootstrap-based measure of support of inferred reticulation events, and using it to determine the number of those events, as well as their placements. A number of samples is generated from the given sequence alignment, and reticulation events are inferred based on each sample. Finally, the support of each reticulation event is quantified based on the inferences made over all samples.

**Conclusions:**

We have implemented our method in the NEPAL software tool (available publicly at http://bioinfo.cs.rice.edu/), and studied its performance on both biological and simulated data sets. While our studies show very promising results, they also highlight issues that are inherently challenging when applying the maximum parsimony criterion to detect reticulate evolution.

## Background

The massive evidence of horizontal gene transfer in prokaryotes and higher organisms and the significant role hybridization plays in speciation of various groups of species in the plant kingdom and beyond, have highlighted the need for developing models and methodologies that augment trees to enable modeling of reticulate evolutionary relationships. Indeed, the computational biology and bioinformatics communities have developed a host of such models and methodologies for reconstructing and evaluating *phylogenetic networks*. Several extensive surveys have been written recently about phylogenetic networks; we refer the reader to [1-7].

One of the most commonly used criteria for reconstructing phylogenetic trees is *maximum parsimony *(MP). Under this criterion, the phylogenetic tree that best fits a sequence data set is one that minimizes the total number of mutations over all possible tree topologies and sequence assignments to internal nodes of the tree topologies. There is a polynomial time algorithm for computing the parsimony length of a fixed phylogenetic tree leaf-labeled by a set of sequences, due to [8], while solving the MP problem (i.e., reconstructing the optimal phylogenetic tree under MP) in general is NP-hard [9,10]. Nonetheless, several heuristics that solve the MP problem efficiently, and with high accuracy, in practice have been devised, such as the ones implemented in the phylogenetic software tool PAUP*[11].

In the early 1990's, Jotun Hein extended the maximum parsimony (MP) criterion to allow for modeling the evolutionary history of a set of sequences in the presence of recombination [12,13]. More recently, Nakhleh *et al*. gave a mathematical formulation of the MP criterion for phylogenetic networks [14], and later studied its performance on biological as well as simulated data sets [15]. The main observation behind defining MP (and other criteria) for phylogenetic networks is that a sequence data set whose evolution involves reticulation events, such as horizontal gene transfer, can be partitioned into smaller, non-overlapping regions each of which has a treelike evolutionary history. Based on this observation, an optimal phylogenetic network under the MP criterion is one that *contains *(*induced*, or *displays*) the set of trees that best fit the evolutionary histories of the smaller regions. More formally, for a set *S *of sequences that can be partitioned into regions *S*^1^, ..., *S*^*k*^, such that each region has a treelike evolutionary history, the parsimony length of a phylogenetic network *N *leaf-labeled by *S *is(1)

where *PS*(*T*, *S*^*i*^) denotes the parsimony length of region *S*^*i *^on tree *T*, where *T *ranges over the set  (*N*) of all trees contained inside network *N*; see [14] for more details. At the lowest level of atomicity, each region contains a single nucleotide, corresponding to the scenario where each site may evolve independently of its neighboring sites. This level of atomicity may be appropriate, for example, for analyzing single nucleotide polymorphism (SNP) data in a population, since, depending on the rate of recombination in the genomic region under study, it may be plausible to have adjacent SNPs "switching" evolutionary histories.

However, in a phylogenetic study involving several species, taking each region to correspond to a single site is unrealistic, and may cause serious problems (such as adding an excessive number of reticulation events to the network so as to fit the evolution of each single site with no homoplasy). In our studies, and given that we seek to find whether a certain gene is horizontally transferred, we take each gene to be a single block. The minimization in Formula (1) indicates that the MP tree, among all trees contained in *N*, is chosen for each region, and the summation implies independence among the regions. In other words, in a phylogenetic analysis, *S*^1^, ..., *S*^*k *^may correspond to *k *loci. In the discussion below, we focus exclusively on the formulations for a single locus (or, a single region).

One of the major challenges of applying the MP criterion to phylogenetic network evaluation and reconstruction is the computational complexity. As phylogenetic trees are a special case of phylogenetic networks, the problem of inferring a phylogenetic network under the MP criterion is NP-hard. Even the problem of computing the parsimony length of a *fixed *phylogenetic network is NP-hard [16]. Nonetheless, the problem of computing the parsimony length of a fixed phylogenetic network is *fixed parameter tractable*, where the parameter is the number of reticulation events (nodes of indegree 2) in the phylogenetic network. Jin *et al*. have provided an array of algorithmic techniques that allow for inferring phylogenetic networks under the MP criterion in a reasonable amount of time [16-18].

A potentially more serious challenge of applying the MP criterion to phylogenetic networks concerns the overestimation of the true amount of reticulation in the evolutionary history of a sequence data set. Based on Formula (1), if *N' *is a phylogenetic network obtained by adding extra reticulation nodes to another network *N*, then *PS*(*N'*, *S*) ≤ *PS*(*N, S*), simply because in this case we have  (*N*) ⊆  (*N'*) (this is Observation 1 in [15]). In other words, under Formula (1), adding extra reticulation nodes to a phylogenetic network either leaves the parsimony length unchanged or improves it; it never makes it worse.

Overestimation of the amount of reticulation in an evolutionary history, then, is inevitable under this formulation of the MP criterion. In particular, given a sequence alignment *S *of *m *sites, with site *i *exhibiting *c*_*i *_states (e.g., 1 ≤ *c*_*i*_*≤ *4 for DNA), a phylogenetic network on which the evolution of each site is *homoplasy free *can be reconstructed. That is, we can infer a network *N *such that

In this paper, we focus on the horizontal gene transfer (HGT) version of the phylogenetic network reconstruction problem, in which a species tree *ST *and a sequence alignment of a gene *S *are given, and a set of edges is sought whose addition yields a network that fits the data under the MP criterion. The *ad hoc *solution to this problem that was adopted by [15] was to observe the improvements in the parsimony length as more HGT events are added, and stop the process when the improvement is below a certain threshold. Such a solution does not provide a systematic way of determining the "right" number of HGT edges. Further, it is not applicable in studies that require a large number of analyses, such as simulation studies. In this paper, we address this problem in a more systematic way. We propose a bootstrap method for estimating the support of an inferred HGT edge, and use that as the basis for a stopping criterion (the bootstrap definition is similar to the one in [19]). Given a sequence alignment *S*, the method generates ℓ sequence alignments with the same dimensions as *S *by sampling (with replacement) sites from *S*, infers HGT edges based on the MP criterion for each sample, and finally assesses the support of each HGT edge based on its frequency in the analysis of all samples. In addition to assessing the support of the placement of an HGT edge, this method can be used to determine when to stop adding such edges.

We have implemented the method in our NEPAL software tool (available publicly at http://bioinfo.cs.rice.edu/), and studied its performance on both biological and simulated data sets. While our studies show very promising results, they also highlight issues that are inherently challenging when applying the maximum parsimony criterion to detect reticulate evolution. In particular, they show that the maximum parsimony criterion may not distinguish among a set of neighboring tree edges, as to which one is the true donor of a horizontal gene transfer event. In this case, we propose a relaxed version of the support formula. Further, we find that resolving non-binary nodes in the species tree, prior to the MP analysis, may help in the accuracy of the inferences made.

## Methods

### Maximum Parsimony of Phylogenetic Networks

**Figure 1:**
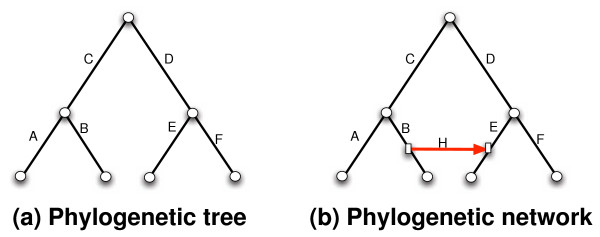
**A phylogenetic tree (a) and a phylogenetic network obtained from it by adding a horizontal edge *H *from edge *B *to edge *E***.

A phylogenetic network is a rooted, directed, acyclic graph, leaf-labeled by a set of taxa, coupled with a set of temporal constraints [20]. In the case of HGT, a phylogenetic network is obtained by adding a set of *horizontal*, or *lateral*, edges to an underlying species tree, where those horizontal edges capture the horizontal transfer events that may have occurred during the evolution of a certain gene under study. More precisely, if *T *is a phylogenetic (species) tree, we obtain a phylogenetic network *N *with a single HGT edge from tree *T *by selecting two edges *e*_1 _= (*u*_1_, *v*_1_) and *e*_2 _= (*u*_2_, *v*_2_) in *T*, splitting each of them, so that these two edges are replaced by four edges , , , , and finally a horizontal edge (*x*_1_, *x*_2_) is added. For example, in Figure 1, an HGT edge *H *is added in this fashion from edge *B *to edge *E *in the phylogenetic tree; the rectangular nodes in the phylogenetic network correspond to the splitting points of the two original edges *B *and *E*. It is important to note that when repeating this process to add other HGT edges, the procedure never splits a horizontal edge.

A tree is *contained *in a phylogenetic network if it can be obtained from the network by the following two steps: (1) for every node in the network, remove all but one of the edges incident into it (i.e., the edges whose head is the node under consideration); (2) for every node *u *with a single parent *p *and a single child *c*, remove *u *and the two edges incident to it, and add a new edge from *p *to *c *(repeat this step as long as such nodes as *u *exist). Given a phylogenetic network *N*, we denote by  (*N*) the set of all trees contained inside *N*.

The parsimony length of a phylogenetic network *N *leaf-labeled by a set of sequences *S *is given by Formula (1) in the Background section, as formulated in [14]. The maximum parsimony problem in the context of phylogenetic networks is, for a given sequence alignment *S*, to infer the phylogenetic network *N *that minimizes *PS*(*N, S*). In this paper, the reticulate evolutionary events we consider are horizontal transfers on individual genes (HGT). In this context, the version of the maximum parsimony problem that we seek to solve is to find for a given (species) tree *ST *and a gene sequence data set *S*, a network *N*, obtained by augmenting *ST *with a set of HGT edges, that minimizes *PS*(*N, S*).

As illustrated in Observation (1) of [15], and reviewed above, this definition of MP on phylogenetic networks does not penalize complexity of the inferred model, instead favoring networks with larger numbers of HGT edges. Two questions arise:

1. When should a method stop adding HGT edges under the MP criterion?

2. How supported are HGT edges that are inferred by the MP criterion?

Combined together, answering these two questions amounts to assessing the significance of a phylogenetic network inferred by the maximum parsimony criterion. To the best of our knowledge, neither of these two questions has been addressed in a systematic way. In the next section, we propose a bootstrap-based method for addressing both questions.

### Inferring Well-supported Phylogenetic Networks

Assume the HGT edge *h *: *X → Y *is inferred by the MP criterion on phylogenetic network *N *and sequence data set *S*. To assess the significance of *h *we generate ℓ sequence alignments, *S*_1_, ..., *S*_ℓ_, with the same dimensions of *S*, by sampling (with replacement) sites from *S*, and for each sequence alignment *S*_*i*_, we redo the calculation of MP on *N *and *S*_*i*_, and record the set *H*_*i *_of all optimal HGT edges inferred. Then, the bootstrap-based support of *h*, *S*(*h*), is calculated as(2)

#### Relaxing the Support Formula: When Ambiguity Helps

**Figure 2:**
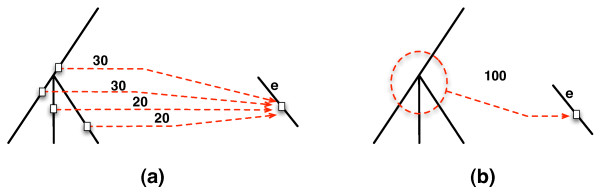
**(a) A scenario where none of four HGT edges identified individually in 100 bootstrap samples has good support (the recipient of each of the four edges is the same node *v *in the species tree)**. (b) When combined, thus allowing for ambiguity in pinpointing the exact source, a well-supported hypothesis of an HGT emerges.

Pinpointing the exact location of an HGT edge is a very hard task in practice, which would be expected to affect the support of inferred HGT. Indeed, our experimental results show that the support of an HGT edge, as given by Formula (2), tends to be very conservative, due to the strict requirement that *h*_*i *_and *h *must be identical (see Results and Discussion section). From our empirical analysis of the performance of MP, we found that the major cause behind a poor support of a correctly inferred HGT edge is that "neighbors" of the source may be as good candidates as the source itself under the MP criterion. We illustrate this in Figure 2. In the cartoon shown in Figure 2(a), four HGT edges, involving edge *e *as the recipient, were identified individually in 100 bootstrap samples, each with the associated support (out of 100). While none of them has good support, combined they produce a well-supported hypothesis of an HGT involving the *clade*, as shown in Figure 2(b). Empirically, we found that this process of introducing ambiguity in the source of an HGT edge often involves immediate neighbor edges of the source. In other words, we can refine Formula (2) of estimating the support of an edge *h *: *D*(*X*) → *Y*, where *D*(*X*) is a set of (neighboring) edges that correspond to potential sources, to obtain(3)

where *H*_*i *_is the set of all optimal HGT edges inferred in the *i*^th ^bootstrap sample. In the case when multiple best HGT edges *H *exist, a support value of *H *is computed as max_*h*∈*H*_(*S*(*h*)).

When Formula (3) is used on the cartoon scenario depicted in Figure 2(a), we obtain an HGT edge with perfect support, whose source is ambiguous, as illustrated with the dashed circle. It is important to note that in biological studies, a group of species, rather than a single specific one, is often reported as the source of a transfer event. This gives further justification for relaxing the formula. In Results and Discussion, we demonstrate the gains obtained by this relaxed formula in analyzing the data of [21]. It is worth mentioning that while our analyses here always revealed ambiguity in the source of an HGT edge, it may be the case that for other data sets there is ambiguity in the recipient as well. In that case, Formula (3) can be extended by using *D*(*Y*) instead and treating it in a similar fashion to the way *D*(*X*) is treated. However, we did not find this to be the case in our analyses, and do not find this surprising.

Replacing HGT edge *h *: *X → Y *by *h'*: *X' *→ *Y *for *X' *∈ *D*(*X*) result in very local change to the topology of the resulting gene tree. On the other hand, replacing *h *: *X → Y *by *h'*: *X *→ *Y' *for *Y' *∈ *D*(*Y*) results in a much greater change to the topology of the resulting gene tree (this depends on how far *X *and *Y *are in the species tree, a measure that we call "diameter" below).

As for how big of a neighborhood *D*(*X*) (or, *D*(*Y*)) one may consider, in our analyses we found that the immediate "neighbors" of an edge are the most relevant. More precisely, if *X *is edge (*u, v*) in the underlying species tree, then *D*(*X*) contains all edges emanating from either *u *or *v*, and the edge incoming into *u*. The reason behind defining the neighborhood *D*(*X*) in this manner is that if an HGT edge *h *: *X *→ *Y *results in improvement *α *to the total parsimony length, then replacing *h *by an edge *h' *: *X' *→ *Y*, where *X' *∈ *D*(*X*), results in an improvement to the parsimony length that is close to *α*.

#### Stopping Criterion

Using the above formulas for bootstrap-based support of an HGT edge, we propose the following procedure for inferring a phylogenetic network under the maximum parsimony criterion starting from a species tree *ST *and a sequence alignment *S *of a gene of interest:

1. **Let ***N *= *ST*.

2. **While ***true*

(a) **Compute **the set *H *of HGT edges, such that for each *h ∈ H*, *PS*(*N *+*h, S*) (*N *+ *h *denotes the phylogenetic network resulting from adding HGT edge *h *to phylogenetic network *N*) is minimum over all networks obtained by adding a single HGT edge to *N*.

(b) Let *b *= max_*h*∈*H*_(*S*(*h*)) and *h' *= argmax_*h*∈*H*_(*S*(*h*)).

(c) if *b *> 70

i. **Let ***N *= *N *+ *h'*.

(d) else

i. **Return ***N*.

In the above procedure, the network is initialized to the given species tree (Step 1). Then, the set *H *of all HGT edges whose addition results in the optimal improvement of the parsimony score is computed (Step 2a). If the maximum support of any edge in *H *exceeds 70 (out of 100), we add the edge and continue; otherwise, we stop adding edges (Step 2c). Hillis and Bull [22] showed that bootstrap values ≥ 70% usually correspond to the "real" clade with very high probability, and this value has been widely accepted as an indication of good support [23]. Below we show that the value 70, as a threshold, works well in practice for the support of HGT edges.

If more than a single locus is involved in the analysis, then we have, as discussed above, a set of sequence alignments *S*^1^, *S*^2^, ..., *S*^*k*^, each corresponding to an individual locus. If these loci have evolved independently, then analyzing each of them individually, using the methodology described above, is sufficient. This may result, for example, in an HGT edge *h *: *X → Y *that has high support based on the analysis of locus *i *and low support based on the analysis of a different locus, *j*. This is not contradictory, since the support of an HGT edge is dependent on the data, and the support should be reported for each HGT edge and each locus independently. Now, let us consider the case when, for example, two loci *i *and *j *are depended (e.g., they are linked). In this case, one could concatenate the sequences from both loci and consider the resulting "supergene" as a single locus in the analysis. This, of course, requires determining if two loci are linked, a question whose treatment is beyond the scope of this paper. Nevertheless, we conjecture that analyzing each gene independently, even when the independence assumption does not hold, may be a safe choice, particularly if enough sites are available for each locus.

### Data Sets

We have implemented the method described above in the NEPAL tool, which is available publicly at http://bioinfo.cs.rice.edu/. Using species trees and sequence alignments of genes from biological and simulated data, we studied the performance of our method in identifying the amount of HGT as well as location of those HGT events in the data sets.

#### Biological Data

We studied 20 out of the 31 mitochondrial gene data sets, which were collected from 29 diverse land plants and analyzed in [21]. These are *cox2*, *nad2*, *nad3*, *nad4(ex4)*, *nad4(exons)*, *nad5*, *nad6*, *nad7*, *atp1*, *atp8*, *ccmB*, *ccmC*, *ccmFN1*, *cox3*, *nad1*, *rpl16*, *rps19*, *sdh4*, and three introns *nad2intron*, *nad5intron *and *nad7intron*. We used a species tree for the data set based on information at NCBI http://www.ncbi.nih.gov and analyzed the entire data set with both seed and non-seed plants together. For each gene data set, we restricted the species tree to those species for which the gene sequence was available. We compared HGTs we have identified with the result of Bergthorsson *et al*. It is important to note that in their analyses, Bergthorsson *et al*. focused only on genes that were horizontally transferred to the (mitochondrial genome of) *Amborella*; in other words, they did not consider HGT events that may not have involved *Amborella*.

#### Simulated Data

We used PhyloGen [24] to generate two 50-taxon species trees *ST*_1 _and *ST*_2 _under the birth-death model. More precisely, we used the following settings for the PhyloGen tool:

birthdeath birth = 1 death = 0 extant = 50

generate replicates = 2

For each species tree, we simulated ten DNA sequence alignments of length 1000 under the Kimura 2-Parameter model, involving HGT events, using the tool of [25]. To achieve this, we used the following settings for the tool:

nb_genes 10

diameter 1. 1.

sampling 100 100

seq_type DNA

seq_length 1000 1000

total_rho 0

total_tau 1

total_rho_prime 0

alpha_l 1.

alpha_s 0.5

subst_model K80

subst_rates 2

We modified the tool of [25] so that it also prints the actual HGT events it simulates. We label the 20 generated gene data sets as *GS*_1_1_, ..., *GS*_1_10_, *GS*_2_1_, ..., *GS*_2_10_. The actual number of HGT events involved in each of the genes is reported in the results' table below.

## Results and Discussion

We have analyzed the biological and synthetic data by applying the procedure given above, to assess the confidence of the postulated HGT edges and determine the number of HGT events by the confidence. For our experiments, we generated 100 sequence alignments by sampling sites with replacement from the original alignment, in all cases for the biological and simulated data analysis.

### Biological Data

**Figure 3:**
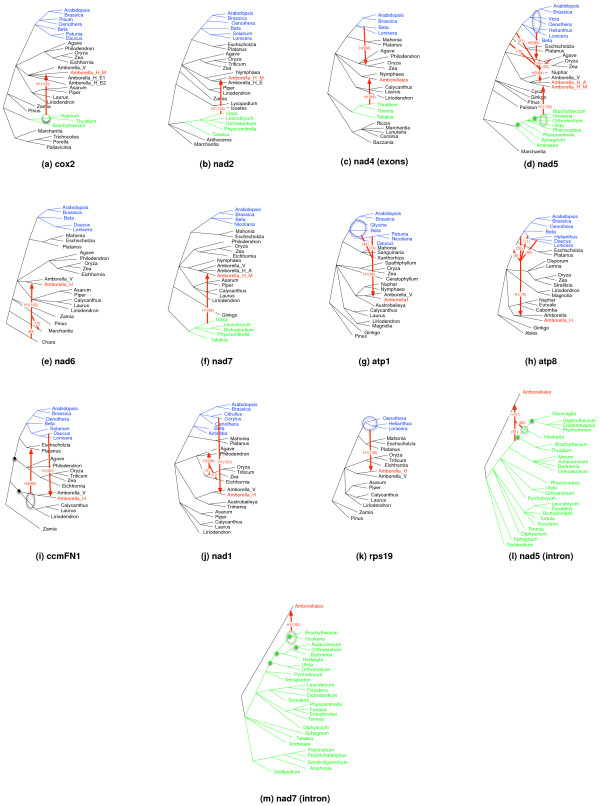
**HGT edges (in red) inferred by the MP criterion, with support values, in parentheses, computed based on Formula (3)**. Ambiguity in the source is denoted by a circle (when drawing a circle was possible) or a multi-source edge. Amborella genes are colored in red, core eudicot genes and moss genes are colored in blue and green. Branch refinements are performed for *nad5*, *ccmFN1*, *nad5intron*, and *nad7intron *at the places marked with solid circles.

The numerical results of analyzing the 20 gene data sets of [21] are given in Table 1, while the inferred phylogenetic networks with strong support for the inferred HGT events for 13 of the gene data sets are shown in Figure 3 (for the other 7 data sets, our method did not identify any HGTs). The three columns under the header [21] in Table 1 correspond to the number of HGTs postulated by Bergthorsson *et al*., the donor group, and support value for each postulated HGT event, as calculated by the test of [26], respectively.

**Table 1:** Mitochondrial gene data sets and HGTs postulated by Bergthorsson *et al*. and those computed by the MP analysis (NEPAL). 'donor' denotes the group from which the gene was transferred (in all cases, the recipient is *Amborella*).

	**Bergthorsson *et al*. **[21]			**MP analysis**					
	
**Gene**	**#HGTs**	**donor**	**SH**	**b1**	**b2**	**b3**	**b4**	**#HGTs**	**F?**
cox2	3	M	<0.001	100	38	-	-	1	Y
		E	NS						Y
		E	NS						
nad2	2	M	<0.001	100	62	-	-	1	Y
		E	NS						Y
nad4(exons)	1	M	<0.001	99	98	44	-	2	Y
nad4(ex4)	1	E	NS	58		-	-	0	Y
nad5	2	M	<0.001	100	95	84	35	3	Y
		A	0.025						Y
nad6	1	B	<0.001	100	26	-	-	1	Y
nad7	2	M	<0.001	99	64	-	-	1	Y
		E	NS						Y
atp1	1	E	0.001	98	33	-	-	1	Y
atp8	1	E	0.008	75	38	-	-	1	Y
ccmB	1	E	NS	39	-	-	-	0	Y
**ccmC**	**1**	**E**	**0.03**	**68**	-	-	-	**0**	**Y**
ccmFN1	1	E	0.004	80	86	37	-	2	Y
cox3	1	A	NS	69	-	-	-	0	N
nad1	1	E	<0.001	100	88	25	-	2	Y
rpl16	1	E	NS	46		-	-	0	Y
rps19	1	E	0.003	100	61	-	-	1	Y
sdh4	1	E	NS	35		-	-	0	Y
nad2intron	1	M	-	66		-	-	0	Y
nad5intron	1	M	-	97	41	-	-	1	Y
nad7intron	1	M	-	100	67	-	-	1	Y

'SH' denotes support of the HGT events as computed by the Shimodaira-Hasegawa (SH) test and reported by Bergthorsson *et al*. (values lower than 0.05 indicate high support, and NS indicates support is not significant). The 'b1', 'b2', 'b3', and 'b4' columns correspond to the support values from Formula 3 for adding the first, second, third, and fourth HGT edges inferred by the MP analysis. Since adding HGT edges stops once a weakly supported edge is encountered, a '-' entry under these columns indicates that adding HGT edges was stopped before. B = Bryophyte, M = Moss, E = Eudicot, and A = Angiosperm.

Bergthorsson *et al*. reported the groups of species to which the donor(s) of horizontally transferred genes belong, rather than the specific donor. In particular, they focused on four groups: Bryophytes, Moss, Eudicots, and Angiosperms. For the recipient, the authors only focused on *Amborella*. Of the 25 HGT events that Bergthorsson *et al*. postulated, 13 were supported, 9 unsupported, and 3 (the 3 intron data sets) had no reported support.

The 'b1', 'b2', 'b3', and 'b4' columns under the MP analysis in Table 1 correspond to the support values from Formula 3 for adding the first, second, third, and fourth HGT edges inferred by the MP analysis. Since adding HGT edges stops once a weakly supported edge is encountered, a dash entry under these columns indicates that adding HGT edges was stopped before. The '#HGTs' lists the number of HGT edges inferred based on the support using the threshold value 70 (see discussion above of the choice of this threshold). In other words, it is the count of non-dash entries minus one in the bootstrap-value columns. The 'F?' column lists in each row whether the HGT postulated by Bergthorsson *et al*. and reported in that row was also found by the MP analysis. The row in gray refers to the case where the HGT postulated by Bergthorsson *et al*. was found by the MP analysis, but with support smaller than 70 (the support of the edge was 68).

Of the 13 HGTs reported in [21] with high support according to the [26] test, the MP analysis with bootstrap supports identified 12, missing one HGT for *ccmC *that has a support value of 0.03 by SH test. While the MP analysis postulated the right HGT edge from the Eudicot group to Amborella (in the sense that the edge resulted in the best improvement in the parsimony length), the bootstrap-based support for this edge was 68, which is lower than the threshold of 70. It is worth mentioning that the SH test reports the weakest support for this case compared to other cases (that are not 'NS'). Further, from the perspective of the parsimony length of the resulting network, postulating the HGT edge for this gene only improves the parsimony length by 6. In other words, this edge has very low support based on all three criteria: parsimony length improvement, bootstrap-based support, and the SH test.

The three HGT edges postulated by Bergthorsson *et al*. for the intron data sets, and which had no support values based on the SH test reported, were all identified by the MP analysis. The HGT edge from the Moss group for the *nad2intron *gene is not well supported, while the HGT edges for the *nad5intron *and *nad7intron *data sets are both strongly supported.

Of the other 9 HGT events reported by the authors with no significant support based on the SH test, the MP analysis identifies seven HGT edges, missing the other two. The identified seven HGTs were all from the Eudictots to *Amborella*, and they were in the *cox2*, *nad2*, *nad4(ex4)*, *nad7*, *ccmB*, *rpl16*, and *sdh4 *data sets. However, none of them is strongly supported according to the bootstrap-based analysis, which is consistent with the SH test results.

In four data sets, the MP analysis identified HGT edges in addition to those reported in [21]. However, none of these edges involved Amborella. One possible explanation for why these edges were not reported in [21] is because the authors focused only on HGT events involving *Amborella*. Another explanation may be the inaccuracy of the parsimony criterion as raised in the preceding section.

Figure 3 shows the phylogenetic networks of 13 of the 20 biological data sets. Each of the HGTs in the networks is marked as 'Hi' representing the *i*-th HGT identified by the MP analysis, and labeled with a bootstrap support value. We used the relaxed bootstrap support value, as given by Formula (3), in 10 out of 13 cases for locating the clade of the source of an HGT since it is hard to identify their exact locations. In 7 cases, clades of the source locations are identified and represented with circles in the figure. Among these 7 cases, *atp1*, *cox2*, *nad5*, *rps19*, *nad5intron*, and *nad7intron *have very high bootstrap support (above 97) for the transfers to Amborella from clades of their source locations. In *cox2*, all three locations inside the circle of 'H1' are identified as equally good sources of an HGT with perfect support. Others show significantly improved bootstrap supports when the sources are identified as a clade instead of an exact location. In some cases, multiple branches with individual bootstrap values labeled are used instead of a clade for identifying more precise source locations of the HGTs. In these cases, the relaxed bootstrap values are marked after the joint points of the branches. Transfers identified by MP but not well supported are not shown in the networks in Figure 3. Refinements, marked with solid circles, are performed for unresolved branches in *nad5*, *ccmFN1*, *nad5intron*, and *nad7intron*, based on MP scores. The MP scores for these four datasets are improved from 927 to 909, from 234 to 227, from 688 to 650, and from 950 to 900 with the marked refinements.

### Simulated Data

The numerical results of analyzing the synthetic gene data sets are given in Table 2. The columns under the 'true HGTs' list the number of HGT edges added by the tool of [25], and *d*1 and *d*2 denote the distance, in terms of the number of branches on the species tree, between the source and recipient of each of the HGT events simulated. When no HGT events are simulated, neither value is provided, and when only one is simulated, only *d*1 is specified. The reason for computing these values is to study the performance of the MP criterion on data sets with varying HGT event *diameters *(the distance between source and recipient), as we hypothesize that as the *diameter *becomes smaller, the performance of the MP analysis may become poorer. An entry with value p* indicates that the diameter is *p*, but that the event is from a branch to another branch that is its descendant in the species tree. While this seemingly contradicts temporal constraints (e.g., that the source and recipient co-exist in time), such a scenario can be explained through extinction or incomplete taxon sampling of taxa; see [20].

**Table 2:** Results of the MP analysis on 20 simulated data sets.

	**true HGTs**			**MP analysis**					
	
**Gene**	**#HGTs**	**d1**	**d2**	**#HGTs**	**F1?**	**F2?**	**b1**	**b2**	**b3**
*GS*_1_2_	0	-	-	0	-	-	15	-	-
*GS*_1_3_	0	-	-	0	-	-	43	-	-
*GS*_1_7_	0	-	-	0	-	-	46	-	-
**GS_1_9_**	**0**	-	-	**1**	-	-	**84**	**45**	-
*GS*_2_1_	0	-	-	0	-	-	27	-	-
*GS*_2_7_	0	-	-	0	-	-	47	-	-
*GS*_2_9_	0	-	-	0	-	-	33	-	-

**GS**_2_2_	**1**	**2**	-	**0**	**N**	-	**55**	**19**	-
**GS**_2_8_	**1**	3*	-	**0**	**N**	-	**24**	**33**	-
*GS*_1_1_	1	4	-	1	Y	-	100	47	-
*GS*_1_6_	1	10	-	1	Y	-	95	45	-
*GS*_1_10_	1	8	-	1	Y	-	76	39	-
*GS*_2_3_	1	7	-	1	Y	-	100	60	-
*GS*_2_4_	1	4	-	1	Y	-	77	38	-
*GS*_2_5_	1	6	-	1	Y	-	100	37	-
*GS*_2_6_	1	9	-	1	Y	-	100	47	-
*GS*_2_10_	1	9	-	1	Y	-	100	15	-

*GS*_1_4_	2	3*	5	2	Y	Y	100	77	13
*GS*_1_5_	2	4*	7	2	Y	Y	100	100	41
*GS*_1_8_	2	5*	7	2	Y	Y	100	98	19

The rows are sorted by the number of true HGTs simulated for each gene data set. The 'd1' and 'd2' columns denote the distance, in terms of the number of branches on the species tree, between the source and recipient of the first and second HGT events simulated, respectively. The 'b1', 'b2', and 'b3' columns correspond to the support values from Formula 3 for adding the first, second, and third HGT edges inferred by the MP analysis. Since adding HGT edges stops once a weakly supported edge is encountered, a '*-*' entry under these columns indicates that adding HGT edges was stopped before.

Under the 'MP analysis', we report the support of the inferred edges as before ('b1', 'b2' and 'b3'), the number of HGT edges detected ('#HGTs'), and whether the true ones were found ('F1?' and 'F2?'), respectively. In this case, a dash entry in the support value columns indicates that the support was not calculated since it was determined already to stop adding HGT edges (i.e., the support for a preceding entry was already < 70).

In this case, for each *GS*_*i_j *_(*i *∈ {1, 2} and 1 ≤ *j *≤ 10), if there are *m *true HGTs, we report the support value of the best *m *+ 1 HGTs inferred by the MP analysis, even if the bootstrap-based stopping criteria indicated stopping the addition of HGT edges at a value smaller than *m*. The rows in gray refers to the cases where the bootstrap-based approach failed to stop with the right amount of HGT.

The results show that when the number of true HGTs, as simulated in the data, is 0, the MP analysis detected no reticulation (or, HGTs) in the data, as the support for adding the first HGT edge is < 70 in all cases with one exception of (*GS*_1_9_). For the cases where the true number of HGTs is 1, there are only two cases where according to the bootstrap-based support no HGTs were postulated, while the correct number of HGTs was postulated in the other eight cases.

It is interesting to note that all cases in which the bootstrap-based method fails to determine the right number of HGT edges have small diameter values. The bootstrap underestimated the true number of HGTs in two cases (*GS*_2_2 _and *GS*_2_8_), inferring incorrectly that the number of HGTs is 0. The horizontal transfer in *GS*_2_2 _and *GS*_2_8 _have diameters 2 and 3, respectively, which are the lowest values among all the simulated data sets. The small diameter of a transfer indicates that the transfer occurred from a branch to another that is almost its immediate sibling or descendant in the species tree. These cases are very hard for the MP criterion to detect, since it detects other HGT edges as yielding the best improvement to the parsimony score. This highlights a fundamental drawback of the MP criterion which is that the HGT edge resulting in the best improvement to the parsimony score is not necessarily the true one. This is not surprising, since MP suffers from similar issues even for reconstructing trees. The second HGT postulated by the MP analysis of the *nad5 *gene differs from that reported by [21] for this very reason: the MP analysis identifies an edge that improves the parsimony score more than the one reported by Bergthorsson *et al*. (the one from *Angiosperm *to *Amborella*).

In the three simulated data sets with two true HGTs, the tool of [25] added one of the two edges from a branch to one of its (not immediate) descendants, making a very hard case for the bootstrap-based support method to detect. However, the MP analysis correctly identifies both HGT edges, and with very high support, in all three cases.

## Conclusions

In this paper, we revisited the maximum parsimony criterion for inferring phylogenetic networks. In previous studies, the criterion was shown to provide very promising results on both biological and simulated data. However, previous work did not provide the means to assess the significance of the number of reticulation events estimated nor the location of the inferred events.

We proposed a systematic measure to serve as a *stopping rule *to the otherwise "overestimating-by-definition" criterion, and demonstrated their performance on 20 empirical data sets and 20 simulated data sets. From the result, it has been shown that bootstrap measure provided very accurate results in general. Further, we found that there are some boundary cases under which the MP criterion performs poorly. Finally, we point out that the bootstrap-based support formula that we presented here can be applied with any method that uses the gene sequences to infer HGT edges, such as maximum likelihood [27].

## Authors' contributions

LN conceived of the study and participated in its design and coordination. HJP and GJ implemented the methods and carried out the experiments. All authors participated in analyzing the results. All authors helped to draft the manuscript, read and approved the final manuscript.
